# DNA-Programmed Chemical Synthesis of Polymers and Inorganic Nanomaterials

**DOI:** 10.1007/s41061-020-0292-x

**Published:** 2020-03-07

**Authors:** Xuemei Xu, Pia Winterwerber, David Ng, Yuzhou Wu

**Affiliations:** 1 Hubei Key Laboratory of Bioinorganic Chemistry and Materia Medica, School of Chemistry and Chemical Engineering, Huazhong University of Science and Technology, Luoyu Road 1037, Hongshan, Wuhan, 430074 People’s Republic of China; 2 Max Planck Institute for Polymer Research, Ackermannweg 10, 55128 Mainz, Germany

**Keywords:** DNA origami, Polymer nanomaterial, Inorganic nanomaterial, Programmed synthesis, Bottom-up nanofabrication

## Abstract

DNA nanotechnology, based on sequence-specific DNA recognition, could allow programmed self-assembly of sophisticated nanostructures with molecular precision. Extension of this technique to the preparation of broader types of nanomaterials would significantly improve nanofabrication technique to lower nanometer scale and even achieve single molecule operation. Using such exquisite DNA nanostructures as templates, chemical synthesis of polymer and inorganic nanomaterials could also be programmed with unprecedented accuracy and flexibility. This review summarizes recent advances in the synthesis and assembly of polymer and inorganic nanomaterials using DNA nanostructures as templates, and discusses the current challenges and future outlook of DNA templated nanotechnology.

## Introduction

DNA, often known as the genetic code, exists in natural organisms. In 1953, the double helix structure of DNA was discovered, which revealed the mystery of life and enabled people to understand clearly the composition and transmission of genetic information [1]. Two reversely parallel complementary DNA single strands can recognize each other via Watson–Crick base pairing, forming a stable DNA double helix with high accuracy from sequence to structure [2]. In the process of life activities, the double helix structure can be dissociated and hybridized dynamically during transcription, replication or repair, which provides a vital guarantee for the recording, transmission and translation of genetic information [3].

In recent decades, enthusiasm for DNA molecules has expanded from the initial biological and chemical arena to that of nanomaterials. Given the advantages of accuracy and design flexibility, advances in DNA self-assembly techniques [4] have made DNA structures a new type of “star” material in nanotechnology. DNA nanotechnology was pioneered by Seeman and co-workers in the 1980s [5–8], and has been revolutionized by Rothemund into a state-of-the-art technology, i.e., DNA origami [9]. This technology allows folding of DNA nanostructures by mixing and annealing a long single circular DNA scaffold with hundreds of short “staple” strands. In practice, this enables the construction of almost any kind of highly complex two-dimensional (2D) and three-dimensional (3D) nanostructure with the aid of computing software such as caDNAno [10–15]. To address the cost limitations of DNA material, Dietz has devised a biotechnological mass production method, which provides impetus for the practical application of DNA origami in the future [16].

**Fig. 1:**
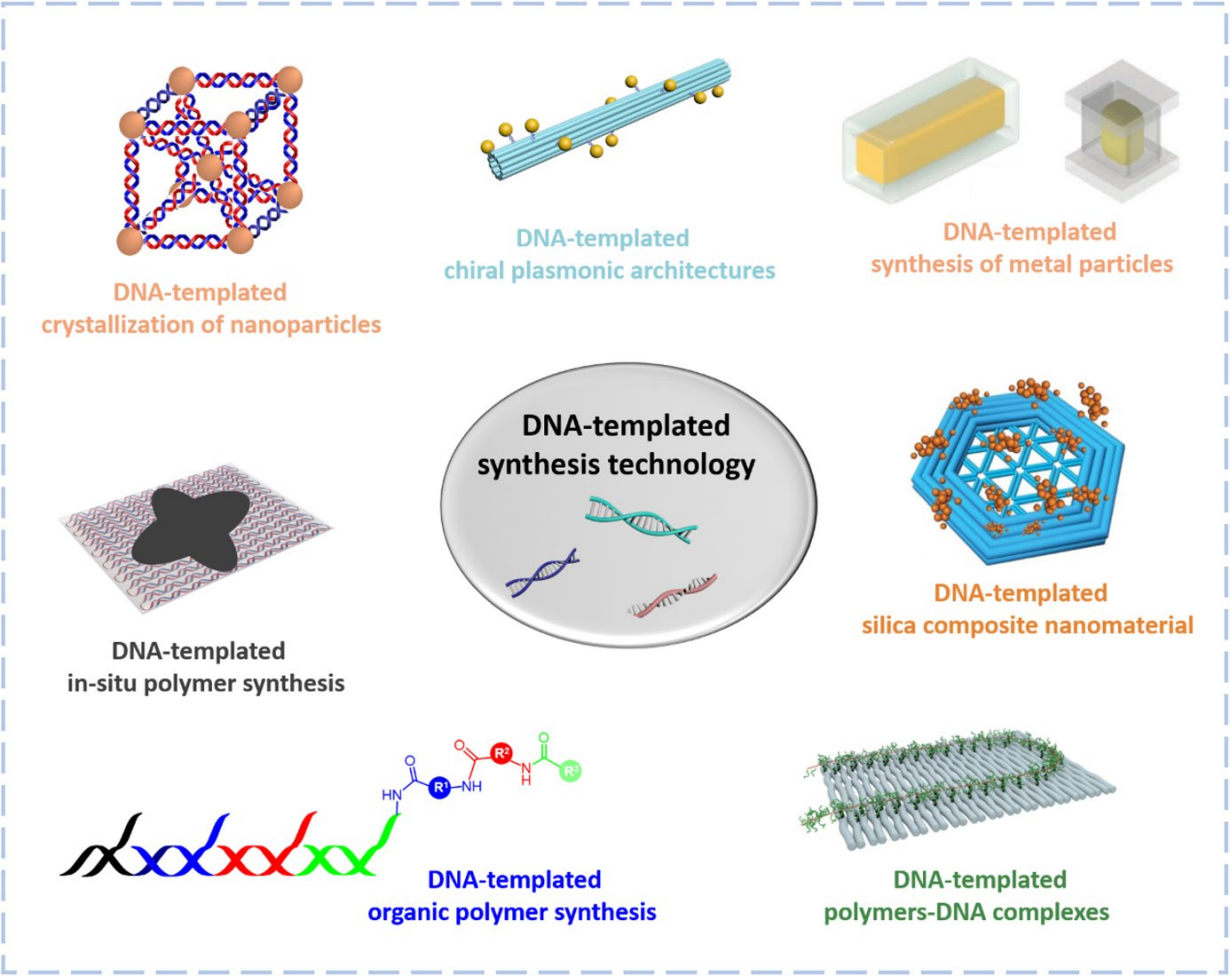
DNA-templated synthetic technology

Intriguingly, the glamour of life is that DNA is not only capable of storing genetic information, but can also precisely translate this information into another material—protein. The sequence-dependent hybridization mechanism ensures that DNA can stably store genetic information and its heredity. In its natural state, DNA provides limited structural features and functional diversity. Therefore, DNA does not directly serve as a functional material, but instead, its sequence-encoded information is transcribed and expanded into the sequence of protein, and, in this way, renders diversity in life. Such an elegant strategy has also inspired material chemists. If DNA nanotechnology could be expanded to prepare diverse materials, the programmable features of DNA nanostructures might endow them with exquisite and unlimited physical and chemical properties, thus bringing a potential revolution to nanomaterial science. Encouraged by this vision, the use of DNA nanostructures as templates to control the precise synthesis of organic and inorganic materials has attracted increasing attention in recent years. In this review, we discuss the latest progress in the field of DNA-programmed material science, with a focus on the synthesis of polymer and inorganic materials employing DNA nanostructures as templates in order to achieve unprecedented accuracy at the nano scale (Fig. 1).

## DNA-Sequence-Encoded Polymer Synthesis

As the most important storage material of natural genetic information, DNA can encode the synthesis of proteins in natural organisms and, accordingly, determine all of life’s activities. The ability to synthesize protein macromolecules using nucleic acids as templates enables proteins to evolve into complex structures and functions with remarkable specificity and reliability. In contrast, traditional chemical polymerization reactions cannot precisely control the sequence of monomers, or their molecular weight distribution. Therefore, synthetic polymers typically could not display defined structures and functions as proteins [17–19]. Despite great progress in controlling the structure [20, 21] and molecular weight distribution [22, 23] of synthetic polymers, it is still difficult to precisely control their sequence and length. However, it has been possible to achieve controlled polymer molecules by taking advantage of DNA sequences to guide the polymerization process. The technology of DNA-encoded polymer synthesis is devoted to more accurate design and preparation of polymer materials, and more precise control of their structure and function [24, 25].

**Fig. 2:**
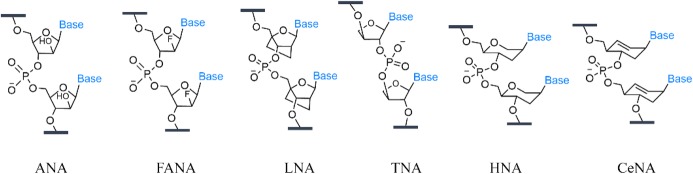
DNA analogs with non-natural backbones that could be enzymatically synthesized based on DNA template

Inspired by the DNA transcription process, Hollige et al. [26, 27] designed a DNA polymerase that allows enzymatic synthesis of nucleic acid analogs with non-natural polymer backbone and DNA hybridizing side chains, such as arabino nucleic acid (ANA) and 2′-fluoro-arabino-nucleic acid (FANA), locked nucleic acids (LNA), threose nucleic acid (TNA), hexonucleic acid (HNA), and cyclohexyl nucleic acid (CeNA) (Fig. 2). These DNA analogs are so-called XNAs. This study demonstrated for the first time that genetic information can be stored in, and recovered from, synthetic genetic polymers not found in nature. They have also shown that some XNAs could be replicated and folded into complex structures. In addition, Chaput and co-workers [28] obtained a thrombin-bound TNA adapter with high affinity and specificity from TNA libraries translated by enzyme-mediated primer extension, demonstrating that TNA has the ability to fold into tertiary structures with sophisticated chemical functions. The enzymatic DNA templating synthesis of synthetic polymer backbone showed therein suggest the potential to obtain synthetic polymers also capable of storing sequence information. However, the type of polymer backbone is highly limited.

**Fig. 3a,b:**
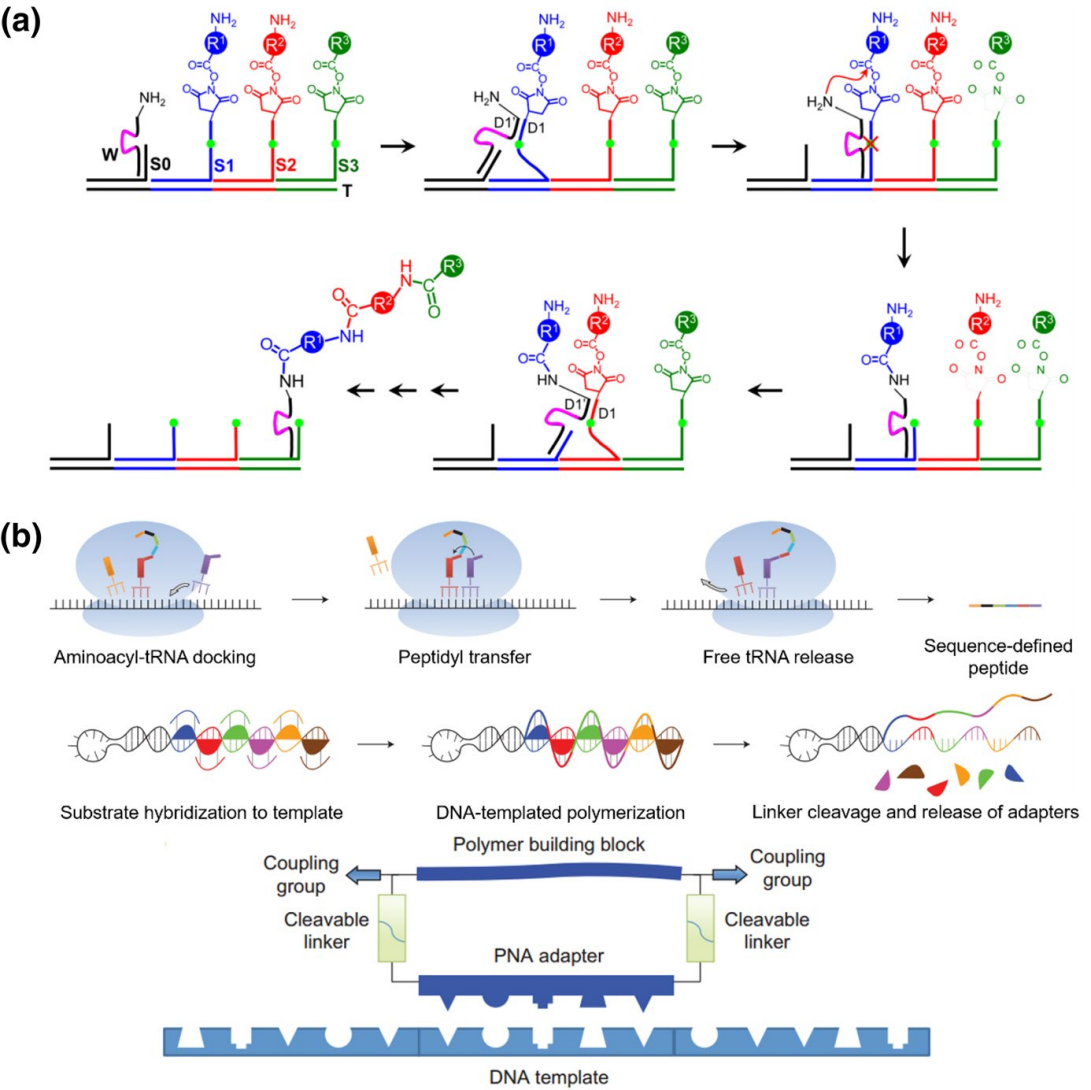
Synthesis of polymers based on DNA templates. **a** DNAsome-mediated multistep synthesis of a triamide product. Reproduced with permission from Ref. [38]. Copyright 2010 Nature Publishing Group. **b** Synthesis of polymers with higher molecular weight arising from the process of translation from DNA into proteins. Reproduced with permission from Ref. [39]. Copyright 2013 Nature Publishing Group

Liu and other researchers also attempted to mimic the principle of the natural gene translation process to achieve chemical synthesis of sequence-controlled polymers based on DNA templates [29–31]. They reported the efficient and sequence-specific polymerization of non-functional [32] and side-chain-functionalized peptide nucleic acid aldehydes [33] with DNA sequence templates, combined with an in vitro translation, functional screening and amplification system [34]. Although these studies showed the potential for chemically mimicking the gene translation process, they are still limited to nucleic acid analogs. Taking a step further, more flexible synthetic monomers could be sequence specifically aligned along the DNA template by conjugating the monomers with short DNA sequences. For instance, Schuster et al. [35] used cyclic and linear DNA structures as template and DNA conjugated 2,5-bis(2-thienyl)pyrrole as monomers to achieve controlled synthesis of highly defined conducting polymers. In addition, Liu’s group devoted intensive effort to study sequentially multistep reactions in a single solution using DNA templates [29–31]. Their early strategy allow DNA-tagged synthetic monomers to sequentially hybridize on a predefined DNA single-strand template, thus initiating spontaneous proximity-driven cascade reactions to connect the monomers into sequence-specific oligomers [36]. Similarly, Turberfield’s group [37] used short single-strand DNA adapters to hybridize two DNA-tagged monomers, thus controlling the reaction sequence, which can also allow orthogonal synthesis of several predefined oligomers in one vessel. Nevertheless, these designs depend highly on the close distance between the assembled monomers to initiate sequential reactions, thus limiting the choice of monomers to very small moieties and hindering reaction efficiency. Thereafter, they further devised an automatic DNA walker that allows programmable multistep organic reactions that better mimic the natural DNA-encoded protein synthesis process. Different monomers were encoded by specific DNA sequences so that they could be hybridized on DNA templates at predefined positions. The DNA walker could then sequentially create an amide bond between these monomers, just like a ribosome forming an ordered polypeptide chain (Fig. 3a) [38]. This strategy can potentially be applied to more diverse type of reactions, and significantly improve synthesis efficiency, particularly for longer sequences. Furthermore, these latter authors mimicked the process of transcription and translation of DNA into proteins in living systems, producing sequence-specific synthetic polymers with higher molecular weight; the structures of these polymers were independent of the DNA templates (Fig. 3b) [39]. These studies clearly exhibited the great potential of sequence-defined polymer synthesis using DNA templates. With this technique, polymers could be anticipated to possess more rationally designed functions and self-assembly behaviors, rather like biomacromolecules. The possibility of synthesizing sequence-defined polymers with diverse functional monomers might open up numerous new opportunities for polymer chemistry, such as precisely controlling dynamic macromolecular recognition, manipulation of sophisticated polymer assembly and creation of smart polymer nano-robots. However, as the aforementioned methods are somewhat tedious and expensive, they serve mainly as a proof-of-concept studies with still limited applications. Therefore, more efficient and facile methods are still required to promote the practical application of DNA-templated polymer synthesis.

## Synthesis of Polymer Nanomaterials Templated by DNA Nanostructures

As an extension of synthetic polymers encoded by simple DNA strands, in recent years, DNA nanostructures with 2D and 3D spatial conformation have also been used as templates for the synthesis of polymer nanomaterials. At present, there are two major objectives for these studies. On the one hand, researchers aim to improve the physical and chemical properties of DNA nanomaterials by polymer modification. Since DNA-folded “nano-robots” have been proposed as ideal intelligent drug carriers at the nanometer scale [40], there have been intensive studies to enhance the stability of DNA nano-robots under various practical application conditions, and to tune their physical and chemical properties by polymer modification. On the other hand, DNA nanostructures can also serve simply as templates to control polymer synthesis and assembly, providing more powerful nanofabrication techniques to achieve exquisite polymer nanostructures.

### Polymer Modified DNA Nanomaterials

**Fig. 4a–d:**
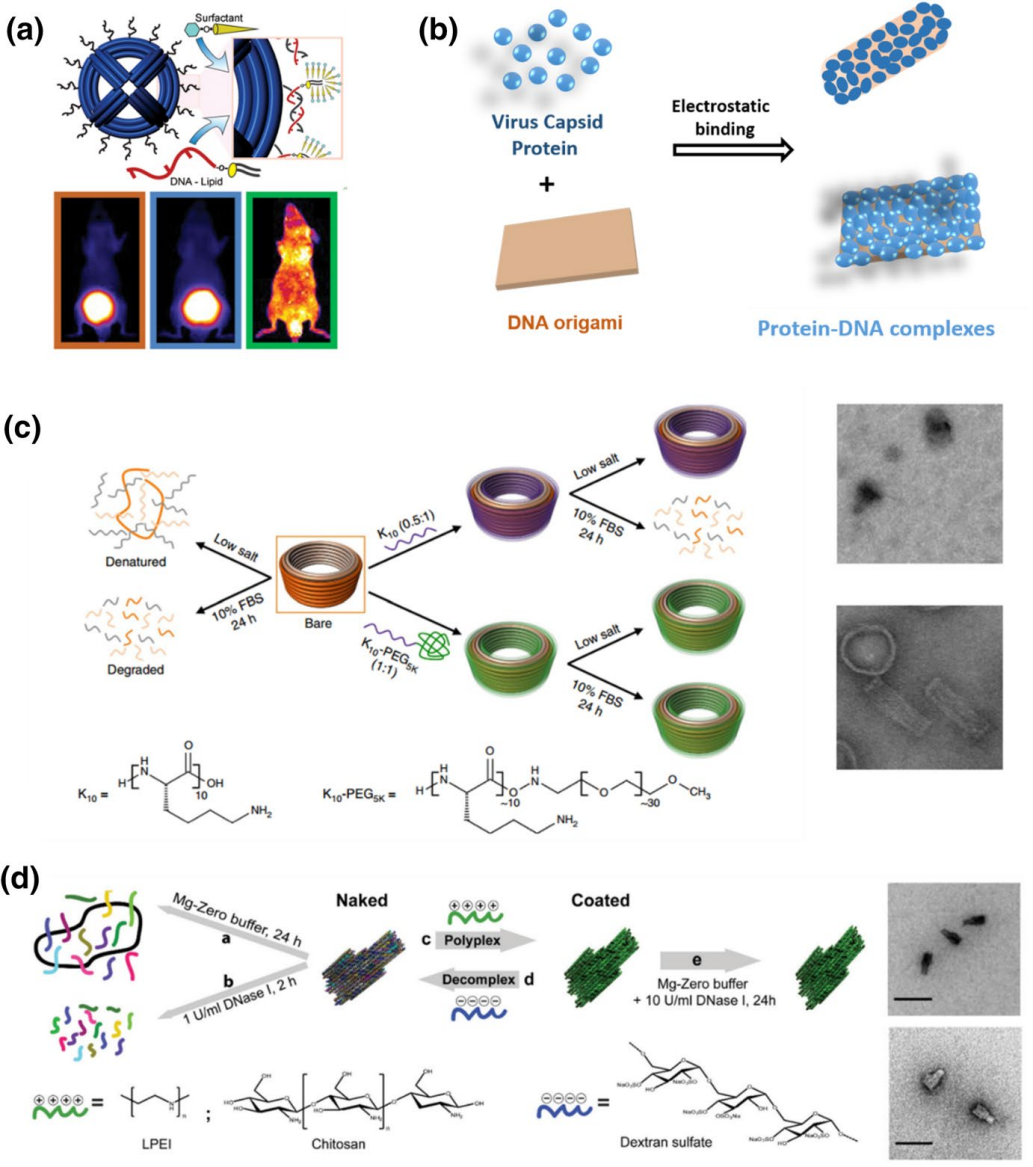
Biopolymers interacting with DNA nanostructures. **a** Liposome membrane encapsulation of DNA nanostructures. Reproduced with permission from Ref. [41]. Copyright 2014 American Chemical Society. **b** Positively charged cowpea chlorotic mottle virus capsid protein encapsulated square DNA origami. Reproduced with permission from Ref. [42]. Copyright 2014 American Chemical Society. **c** Electrostatic adsorption between synthetic polymer and DNA nanostructure template. Reproduced with permission from Ref. [46]. Copyright 2017 Nature Publishing Group. **d** Reversible assembly of synthetic and natural cationic polymers with DNA nanostructures. Reproduced with permission from Ref. [47] Copyright 2018 Royal Society of Chemistry

Various types of substances have been used to encapsulate DNA nanostructures to improve their physical and chemical stability. For instance, by immobilizing lipid-modified DNA on the nanostructure surface, liposome can be formed around the DNA nanostructure to enhance cell uptake efficiency (Fig. 4a) [41]. However, this method requires pre-modification of the DNA nanostructure, and it is tricky to avoid competitive micellar formation by amphiphilic DNA-lipid conjugates. Thereafter, Kostiainen and co-workers studied a series of positively charged materials designed to wrap the negatively charged DNA nanostructures via simple electrostatic interactions. In this regard, the naturally positively charged cowpea chlorotic mottle virus capsid protein was first tested and found to be beneficial to increase the efficiency of DNA nanostructures entering cancer cells (Fig. 4b) [42]. In addition, they also designed synthetic polymer conjugates with a positively charged block to interact with DNA and an uncharged hydrophilic block to facilitate aqueous stability and biocompatibility. They showed that both the positively charged PDMAEMA-PEG copolymer and the dendron-conjugated bovine serum albumin (BSA) could provide the expected functions for encapsulating brick-shaped DNA nanostructures [43, 44]. The protected DNA nanostructures exhibited complete resistance to DNase degradation, and their cell uptake efficiency was significantly enhanced by more than 2.5 times [44]. These outcomes were consistent with reports from others. For instance, Schmidt and co-workers [45] showed that polyethylene glycol and polylysine copolymers (PEG-PLys) can protect the structure of DNA against DNase I degradation and improve stability in low ionic strength buffer. Shih’s group also reported that PEG-PLys copolymers with different molecular weights have dissimilar impacts on the stability of DNA origami [46]. These conjugates confer >1000-fold increased stability against digestion by serum nucleases. Particularly, PEG-PLys copolymer-encapsulated DNA nanostructures can survive uptake into endosomal compartments and, in a mouse model, exhibit a modest increase in pharmacokinetic bioavailability (Fig. 4c). Similarly, Barišić and co-workers reported that the cationic polysaccharide chitosan and synthetic linear polyethyleneimine (LPEI) can also protect the structural integrity of DNA origami (Fig. 4d) [47]. A similar effect was observed when using a chemically modified protein—cationic human serum albumin (HSA)—that is more biocompatible and easily available [48]. These investigations clearly demonstrated that decoration of DNA nanostructures with cationic polymers could be an efficient way to improve their physiological stability and modulate their in vitro and in vivo distribution. In this way, DNA nanostructures can better serve as smart drug delivery carriers for broader biomedical applications. However, it is noteworthy that some essential features of DNA nanostructures, such as their precisely positioned surface functionalities, DNA-sequence-dependent molecular recognition, and the intelligent mobility of specially design DNA robots, were lost during this encapsulation process. Therefore, future studies are expected to pay more attention to controlled polymer modification of DNA nanostructures at only specific positions to enhance their stability and biocompatibility while maintaining functionality. Some methods presented in the following section on Bottom-up Fabrication of Polymer Materials might provide such opportunity, but their potential in this aspect has not yet been fully explored.

### Bottom-up Fabrication of Polymer Materials

**Fig. 5a–d:**
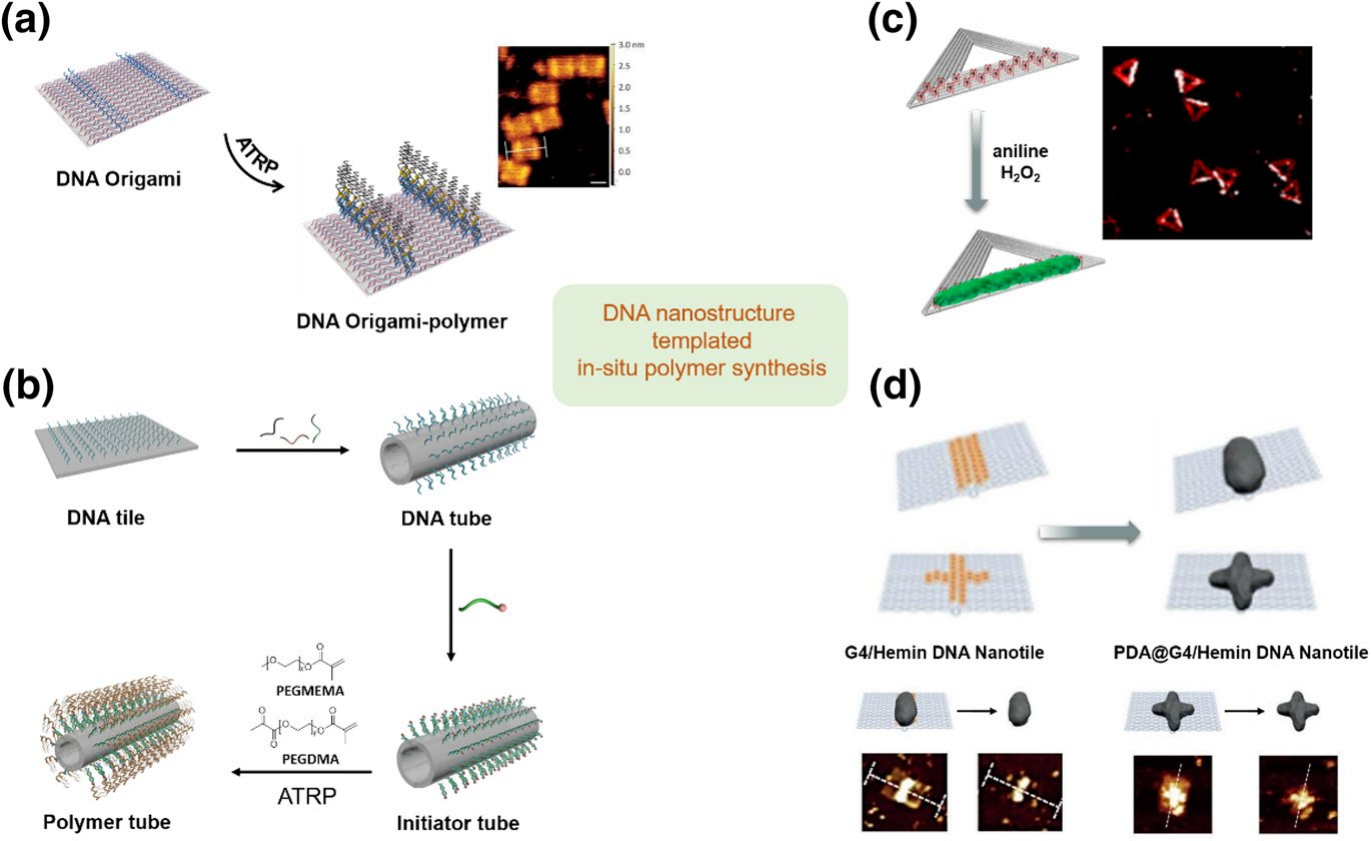
In situ synthesis of DNA nanostructure templated polymers. **a** Bottom-up fabrication of polymers on DNA origami template by in situ atom transfer radical polymerization (ATRP). Reproduced with permission from Ref. [50]. Copyright 2016 Wiley-VCH. **b** Polymeric shell on DNA origami template for enhancing the stability of DNA materials. Reproduced with permission from Ref. [51]. Copyright 2018 Royal Society of Chemistry. **c** Shape-controlled conductive polyaniline on DNA templates. Reproduced with permission from Ref. [53]. Copyright 2014 American Chemical Society. **d** Shape-controlled nanofabrication of polydopamine on DNA templates Reproduced with permission from Ref. [54, 55]. Copyright 2018 Wiley–VCH

Apart from their attractive properties and as yet not fully exploited potential as drug carriers, a role for DNA origami nanostructures as templates to control the growth of polymer nanomaterials has also been envisioned. Conventional synthetic polymerization reactions in liquid phase normally result in randomly entangled polymer chains. In contrast, by combining traditional polymer synthesis methods with DNA strands or nanostructures based on site-specific modification strategy, pre-designed polymer nanomaterials were successfully obtained. This could be achieved by in situ atom transfer radical polymerization (ATRP) polymerization on DNA chains—a technique established by Matyjaszewski and coworkers [49]. By modifying a DNA strand with a ATRP initiator, polymers can grow directly from the 5′-terminus of a DNA chain [49]. Based on this method, Weil and Wu and colleagues [50] achieved the synthesis of polymer nanomaterials with unique shape and patterns by pre-positioning of initiators on a rectangular DNA origami tile. In this regard, the DNA tile can be considered as a “screen” with around 200 sequence-encoded “pixels”. A single-strand sticky end was placed at the “pixel” that was desired to grow polymer later on. Thus, ATRP-initiator-modified DNA sequences could be hybridized on the sticky ends, thereby allowing an in situ polymerization reaction from the immobilized initiators (Fig. 5a). In this way, polymers with a designed nanopattern could be synthesized precisely. Moreover, polymer nanostructures could be released from the DNA template simply by heat-induced disassembly of the DNA tile. Compared with traditional lithography and self-assembly-based polymer nanomaterial preparation techniques, this method features high precision (up to a few nanometers) and efficiency (one-pot reaction in solution), and brings new opportunities in the synthesis of polymer nanomaterials. On the basis of this work, Weil and Wu and co-workers extended this in situ polymerzation method from 2D DNA origami to its 3D counterpart [51]. The rectangular DNA origami was folded into a tube structure, and a controlled ATRP reaction was realized on the outer surface of the tube to construct a polymer-protecting shell. Meanwhile, the inner cavity of the tube is still available for further modification to accommodate other reactions (Fig. 5b). Notably, the defined polymer modification significantly enhanced the stability of DNA nanotubes, similar to that observed with the polymer encapsulation methods discussed above in the section on  Polymer modified DNA nanomaterials [51]. Moreover, the functional positions (such as the inner cavity) on DNA nanostructures could remain unaffected, which could be further modified with guest molecules. Therefore, this method holds great potential for drug delivery, with more opportunities to tune the in vivo behaviors and design complex functionalities.

In addition to controlled radical polymerization, DNA template technology can also be applied to the synthesis of other patterned polymers. Ding’s group reported the catalytic polymerization of aniline on DNA nanostructures [52, 53]. In the presence of hydrogen peroxide, conductive polyaniline could be synthesized at the desired position on double-stranded DNA [52] and on triangular DNA origami nanostructures via a pre-immobilized horseradish peroxidase- or hemin-catalyzed polymerization reaction (Fig. 5c) [53]. This method of preparing conductive polymers in a controllable manner on DNA origami provides a new strategy for the design of nanocircuits. Based on this localized catalysis concept, Weil and co-workers reported shape-controllable in situ polydopamine synthesis on DNA nanostructures by positioning G-quadruplex groups as designed on a rectangular DNA tile and incorporating hemin as a cofactor [54, 55]. In the presence of hydrogen peroxide, the G-quadruplex/hemin DNAzymes mimic horseradish peroxidase activity and induce the oxidative polymerization of dopamine with predesigned patterns [54]. Alternatively, photoirradiation can also initiate the dopamine polymerization reaction in this system [55]. This methodology renders it possible to construct anisotropic polydopamine nanostructures such as nanorods and nano-crosses, which are difficult to achieve by conventional synthesis. These authors further demonstrated that the synthesized polydopamine nanostructure could be retained after removing the DNA template (Fig. 5d), therefore providing a new strategy for polydopamine nanofabrication.

**Fig. 6:**
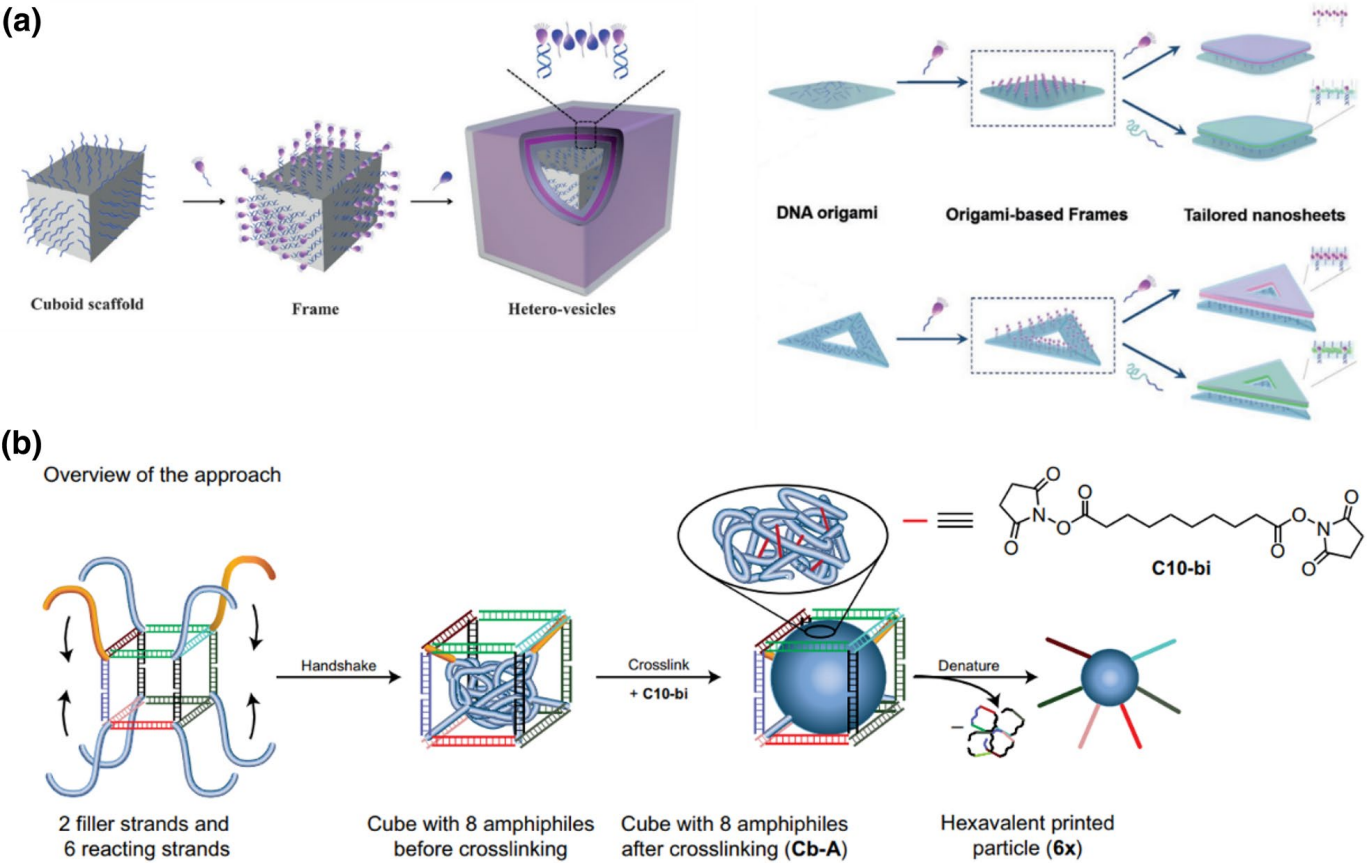
Morphology control of polymersomes on DNA nanostructures as scaffolds. **a** DNA origami mediated “frame guided assembly”. Reproduced with permission from Ref. [56, 57]. Copyright 2017 and 2016 Wiley-VCH. **b** Formation of hydrophobic polymer nanoparticle in DNA templates. Reproduced with permission from Ref. [58]. Copyright 2017 Nature Publishing Group

In addition to DNA nanostructure-templated in situ polymer synthesis, Liu and colleagues have developed a series of methods to use DNA nanostructures as scaffolds to control the morphology of polymersomes and liposomes, which have been collectively named as the “frame guided assembly” technique. It is well known that spontaneously self-assembled vesicles of amphiphilic polymersomes and liposomes are usually spherical. However, Liu’s methods allowed construction of cubic shaped polymersomes and nanometer liposome sheets depending on the DNA template used. Hereby, they could even overcome the surface tension force in nature (Fig. 6a) [56, 57].

Other than controlling the shape and morphology of polymers, DNA templates allow the installation of functional handles on defined positions of polymer nanoparticles. Toward this end, Sleiman and colleagues reported a method of synthesizing DNA-imprinted polymer nanoparticles with monodispersity and prescribed DNA-strand patterns inside a DNA cage (Fig. 6b) [58]. They first immobilized DNA-polymer conjugates on predefined positions of the DNA cage, and then crosslinked the polymers inside the cage. In this way, once the DNA cage is decomposed, DNA handles pre-conjugated with polymer precursors are left on the crosslinked polymer cores with predesigned geometries. This is the first method to achieve nanoparticles with exact number and orientation of asymmetric modifications, which could significantly advance the area of polymer assembly. The works described above strongly indicate the unique advantages of DNA nanotechnology for controlling polymer morphology and function. The features achieved by these methods are almost impossible to achieve with conventional polymer synthesis, and are therefore particularly attractive for preparation of exquisite polymer materials for advance applications, such as intelligent polymer nanorobots, elaborate biosensors and intricate soft electronic devices.

### Routing of Single Polymer Chain

**Fig. 7a–c:**
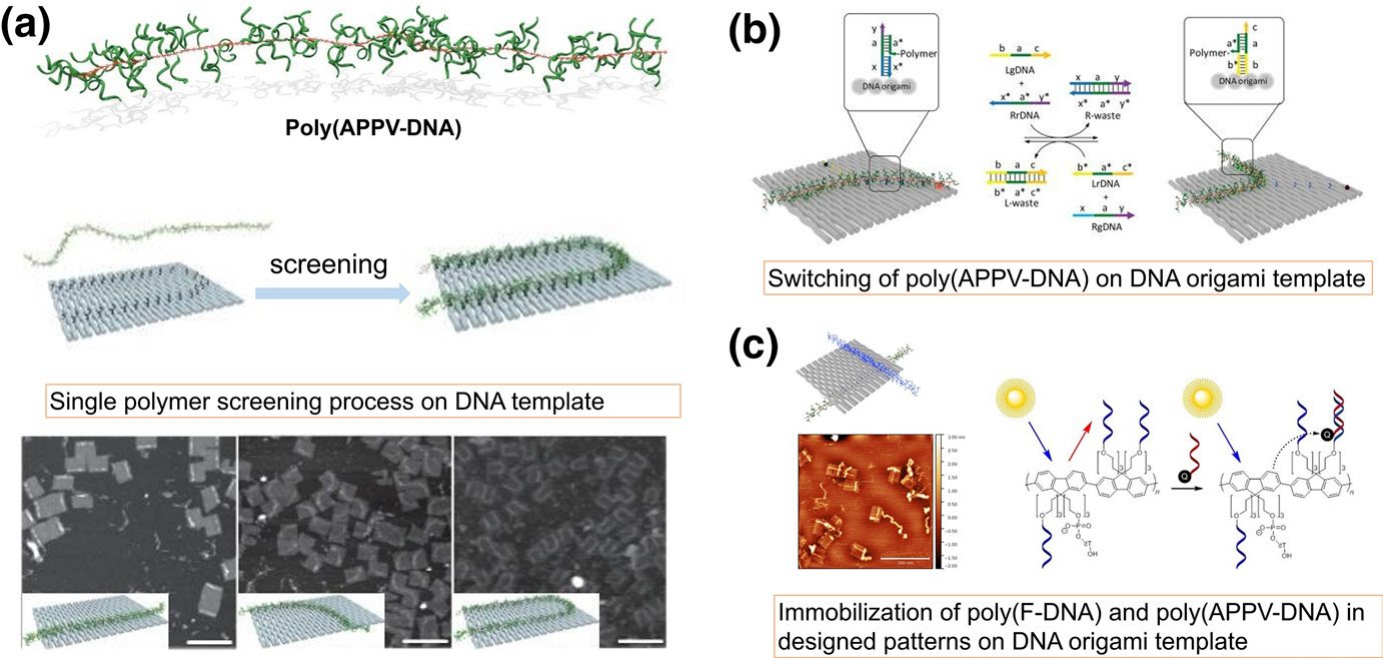
DNA-nanostructure-templated synthesis of single synthetic polymers. **a** Single polymer screening process based on DNA origami templates. Reproduced with permission from Ref. [59]. Copyright 2015 Nature Publishing Group. **b** Programmed switching of single polymer conformation on DNA origami template. Reproduced with permission from Ref. [60]. Copyright 2016 American Chemical Society. **c** Single polymer manipulation and energy transfer investigation of poly(F-DNA) conjugated polymer. Reproduced with permission from Ref. [62]. Copyright 2016 Wiley-VCH

In addition to controlling the morphology of polymer nanomaterials, DNA nanostructures can even be used to manipulate the molecular conformation of an individual polymer chain. Gothelf and colleagues prepared synthetic polymer wires containing short oligonucleotides that extend from each repeat [named poly(APPV-DNA), see Fig. 7a]. The oligonucleotide side chains allow the polymer wire to assemble on the desired positions of DNA nanostructures where the complementary sticky sequences were pre-allocated. In this way, a single polymer chain could be picked up from the polymer solution and immobilized on a DNA template following a predesigned route (Fig. 7a) [59]. Moreover, they can even achieve controlled conformational switching of a single polymer chain on the DNA nanotile based on toehold-mediated strand displacement (Fig. 7b) [60]. The controlled aggregation of poly(APPV-DNA) polymers in solution through varying the ionic environment and sequence-specific DNA interactions was demonstrated in their recent work [61].

On the basis of these works, the team developed a novel hybrid DNA–polyfluorene material, poly(F-DNA), wherein the backbone of polyfluorene was a conjugated polymer with special optical and electronic properties. The fluorescence emission of poly(F-DNA) could be quenched efficiently upon binding to very small amounts of complementary DNA carrying a small molecule quencher. Furthermore, they showed controlled energy transfer between poly(F-DNA) and poly(APPV-DNA) mediated by Watson–Crick base pairing (Fig. 7c) [62]. This concept opens up possibilities for studying the molecular interactions between polymers with different structures via intramolecular energy transfer. These studies clearly demonstrate that DNA nanotechnology provides unprecedented opportunities for handling individual synthetic polymer chains, thus revealing immense prospects for studying and utilizing the single-molecule properties of polymers. These designer organic polymer–DNA complexes are expected to be used as single molecular wires to support the design of high-precision nanocircuits in the future.

## Assembly of Inorganic Nanoparticles Based on DNA Nanostructures

From the perspective of chemical structure, the DNA strand is an organic macromolecule composed of C, H, O, N, and P. Among the virtues of DNA molecules are their controllable structure, adjustable sequence and ease of modification. However, they lack the mechanical strength and quantum optical properties that are commonly available in inorganic nanomaterials. On the other hand, it remains challenging for conventional synthesis of inorganic nanoparticles to achieve arbitrary shapes and controllable assembly, which are highly important in achieving the desired optical and mechanical properties. Therefore, combining the benefits of both materials, DNA-assisted inorganic nanoparticle synthesis and assembly presents an attractive way to create rationally designed nanomaterials with unique features. In this context, modifying nanoparticles (Au, Ag, etc.) with oligonucleotides was found valuable to create nanoparticle crystals with the desired lattice form. DNA nanostructures could even serve as molds to control the morphology of inorganic nanoparticles during synthesis. Moreover, using DNA nanostructures as templates could even fabricate chiral plasmonic metamolecules. This section will summarize recent developments in this area.

### DNA-Programmed Nanoparticle Super-Lattice Crystallization

In 1996, the groups of Mirkin and Schultz published two articles in* Nature* at the same time that revealed the possibility of using oligonucleotide-conjugated gold nanoparticles to guide the formation of nanoparticle assembly [63, 64]. DNA oligonucleotide chains with end-caped sulfhydryl groups were modified on gold nanoparticles (AuNPs) by Au–S bonds, while the reversible assembly of gold nanoparticles was controlled by adding designed DNA linkers with complementary sequences. When DNA duplexes with complementary sequences on both sides were added, the AuNPs formed aggregations with regular interspaces [63]. When the complementary oligonucleotides were positioned at the desired distances along the DNA duplex, defined AuNP dimers and trimers with controllable spacing could be achieved [64]. Since then, the self-assembly of inorganic nanoparticles templated by DNA oligonucleotide chains has advanced rapidly, and has found broad application in the construction of diagnostic tools for nucleic acid, protein and bacteria [65–69], as intracellular probes [70–73], and as selective detection biosensors [74–76] and gene regulators, etc. [77].

**Fig. 8a–c:**
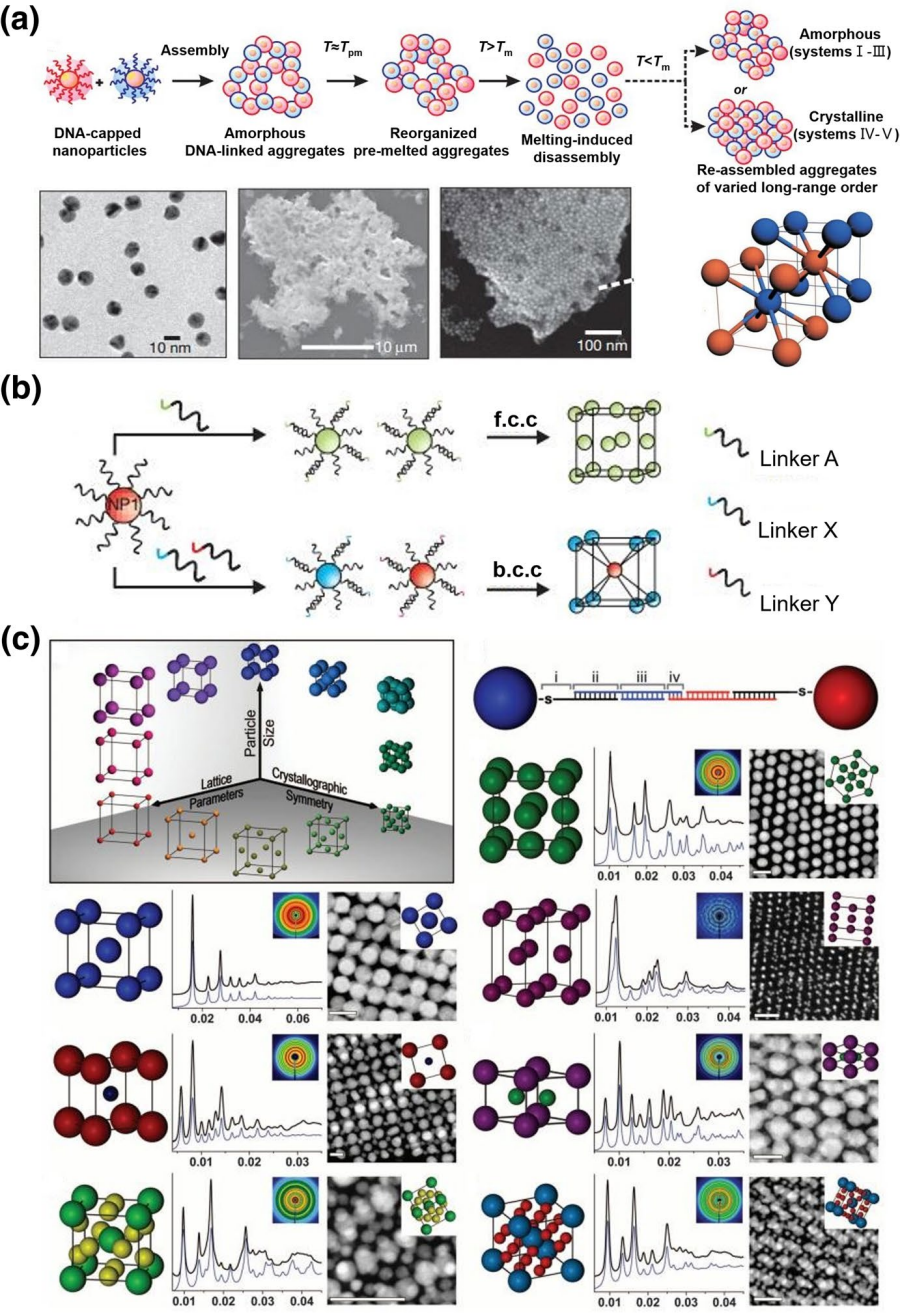
DNA-strand-guided crystallization of inorganic nanoparticles. **a**, **b** The length of the DNA strand and the size of gold nanoparticles influence the crystal pattern. Reproduced with permission from Refs. [78] and [79]. Copyright 2008 Nature Publishing Group. **c** Various crystal lattice structures and their characterization based on DNA-guided assembly of gold nanoparticles. Reproduced with permission from Ref. [80] Copyright 2011 American Association for the Advancement of Science

Despite the attractive applications of these small nanoparticle aggregates, preparation of perfect macroscopic nanoparticle crystals with super lattices was a long-standing challenge until 2008 when the groups of Gang and Mirkin independently reported DNA-guided gold nanoparticle crystals (Fig. 8a, b) [78, 79]. They demonstrated that the growth and assembly of gold nanocrystal structures could be controlled by changing the length of DNA strands between gold nanoparticles. Lattice structures could be accomplished through precisely designing the base sequences and lengths of DNA strands. The Mirkin group subsequently engaged in the manipulation of gold nanoparticles using DNA chains to investigate more diverse crystal superlattices (Fig. 8c) [80]. Moreover, not only were various self-assembled structures from a single inorganic nanoparticle to a superlattice achieved [81–87], the properties of the assembled structures, such as temperature-dependent crystal topology [88], crystal surface energy [89], and dynamic DNA strand substitution [90], were also studied in detail. The mechanism of DNA-templated nanoparticle self-assembly was explained by the repulsive force between colloids [91] and the enthalpy change [92]. The dynamic properties of DNA-templated nanoparticle crystallization [93], in conjunction with previous works [94, 95], revealed that the number of DNA chains per nanoparticle and temperature were the main factors controlling the interaction strength between nanoparticle building blocks.

All the examples mentioned above require gold nanoparticles pre-modified with DNA linkers. Recently, Kostiainen and colleagues reported that ordered 3D gold nanoparticle superlattices could be accessed through sole electrostatic interactions between positively charged gold nanoparticles and negatively charged DNA nanostructures [96]. This approach provided an easier alternative to create a nanoparticle crystal lattice when the accurate design of different types of lattice structure is not required.

These studies realized programmable synthesis of macroscopic materials from rationally designed microscopic nanoparticles, thus providing unprecedented control over the microstructures of bulk materials. Conventional material synthesis generally results in modest control over the placement of, the periodicity in, and the distance between, particles within the assembled material. DNA-assisted strategies allow nanoparticles to be assembled in a designed manner with high precision, which is essential for the design of metamaterials with superior properties in the future.

### Inorganic Nanoparticle Growth with Controllable Dimensionality

**Fig. 9a,b:**
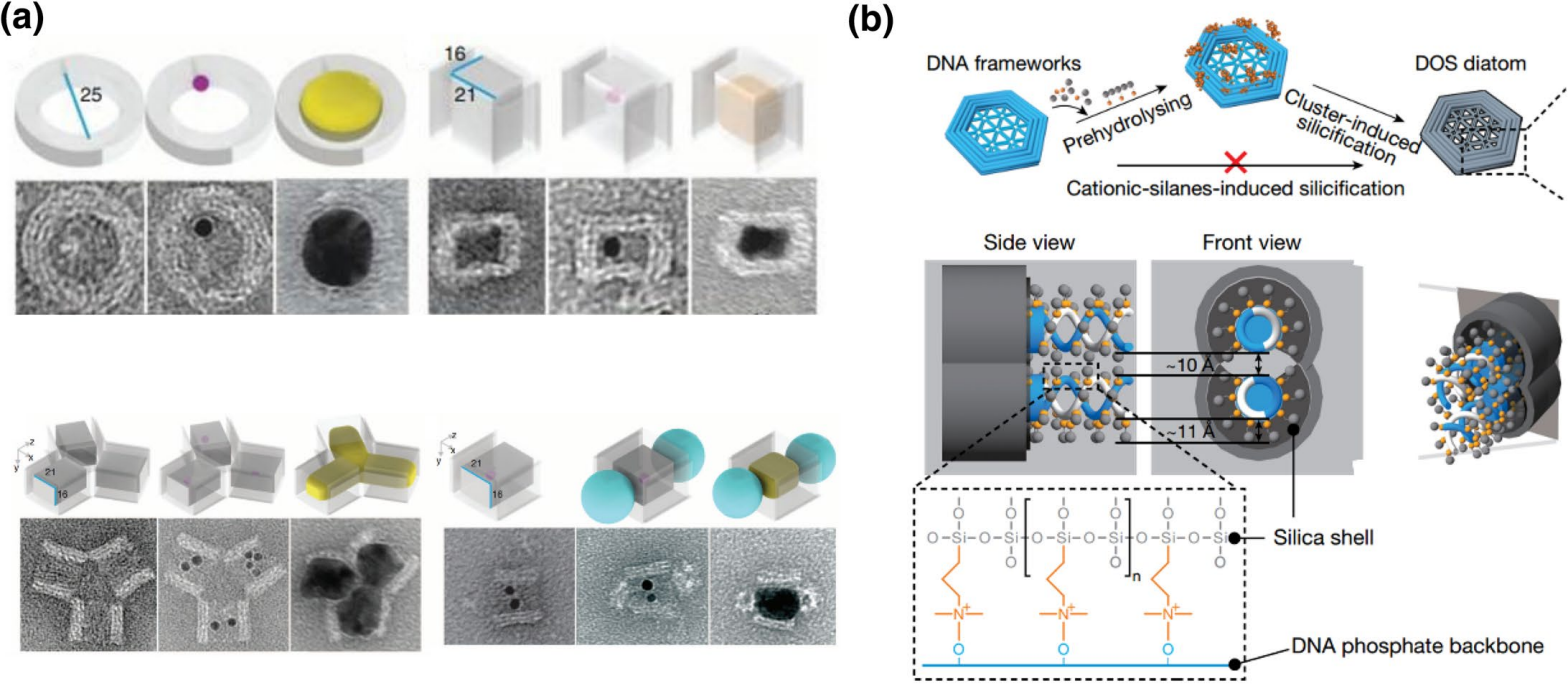
DNA-nanostructure-templated casting growth of metal nanoparticles with controllable dimensionality. **a** Casting metal nanoparticles with predesigned 3D shapes based on DNA nanostructure templates. Reproduced with permission from Ref. [97]. Copyright 2014 American Association for the Advancement of Science. **b** Complex silica composite nanomaterials templated by DNA origami. Reproduced with permission from Ref. [100]. Copyright 2018 Nature Publishing Group

In addition to guiding the assembly of pre-synthesized nanoparticles, DNA nanostructures could also serve as a mold to confine crystal growth during nanoparticle synthesis. In 2014, Yin and co-workers proposed the strategy of casting the growth of inorganic nanoparticles with controllable shapes inside DNA nanostructures cavities. Small gold nanoparticle seeds modified with DNA strands were hybridized onto sticky ends inside the origami cavity. Thereafter, gold nanoparticles were grown in the DNA cavities to completely fill the space. Various shapes of silver or gold nanoparticles can be obtained by designing DNA origami with the apppropriate cavity structures. Moreover, composite inorganic nanoparticles were also accessible by introducing two quantum dots at both ends of the cavity (Fig. 9a) [97]. Analogously, Seidel and colleagues used the DNA origami cavity as a mold to control the growth of gold nanoparticles, and this process was successfully observed by TEM spectroscopy with stepwise addition of chloroauric acid [98, 99]. Inspired by these studies, Fan and Yan and their coworkers recently also presented a general method for creating biomimetic complex silica composite nanomaterials based on 1D, 2D, and 3D DNA nanostructures ranging in size from 10 to 1000 nm (Fig. 9b) [100]. Silicification was achieved through mixing DNA origami templates with prehydrolyzed TMAPS [*N*-trimethoxysilylpropyl-*N*,*N*,*N*-trimethylammonium chloride and TEOS (tetraethyl orthosilicate)], thus a silica shell was formed on DNA origami templates. This represents the first protocol to access highly sophisticated but flexibly designed silica oxide nanostructures that could not be realized by conventional silica nanofabrication techniques. The feasibility of this method was also demonstrated in a parallel study by Heuer-Jungemann [101], which collectively revealed the charm of DNA-templated synthesis to achieve various materials with ingenious nanostructures.

### Chiral and Plasmonic Arrangement of Inorganic Nanoparticles

On the basis of controlling the crystallization and in situ growth of gold nanoparticles, DNA-templated synthesis and assembly techniques can also manipulate the assembly behavior of other diverse inorganic nanoparticles, and even change their inherent physical and chemical properties. It is known that when metal particles are placed in close proximity, their particle plasmons (collective oscillations of conduction electrons) become coupled and induce numerous interesting optical phenomena [102–104]. However, in practice, it is a challenge to ensure that the spacing between two nanoparticles can be arranged as closely as predesigned. As a fully addressable, easily functionalized platform, DNA nanostructures have been used recently to control the spatial organization of discrete nanoparticles with nanometer accuracy [105]. Nanoparticles can be arrayed in any nanometer distance and space on DNA nanostructures with defined patterns. For example, left-handed and right-handed arrangements of gold nanoparticles and nanorods on DNA nanostructures have been prepared to study chiral plasmonic properties at the nanoscale. Furthermore, a variety of chiral plasmonic metal–organic nanostructures [106–108] and chiral colloidal liquid crystals [109] are accessible via DNA- templated nanotechnology.

**Fig. 10a–g:**
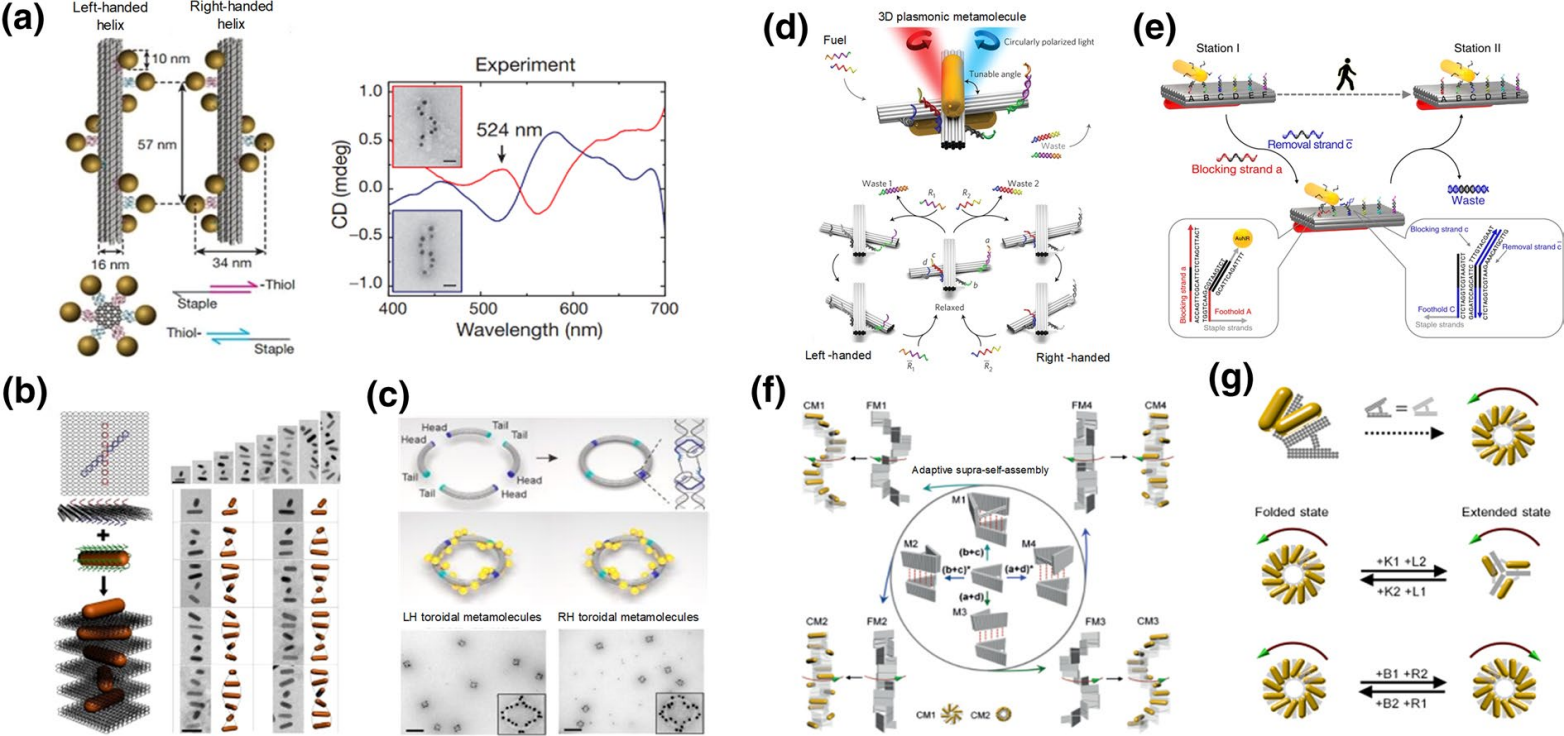
DNA-nanostructure-templated arrangement of nanoparticles with chiral plasmonic properties. **a** Left- and right-handed arrangement and circular dichroism (CD) spectra of gold nanoparticles (AuNPs) on rod-like DNA origami template. Reproduced with permission from Ref. [111]. Copyright 2012 Nature Publishing Group. **b** Anisotropic gold nanorod (AuNR) helical superstructures based on DNA origami sheets. Reproduced with permission from Ref. [112]. Copyright 2015 American Chemical Society. **c** Plasmonic toroidal metamolecules assembled by a DNA origami template. Reproduced with permission from Ref. [113]. Copyright 2016 American Chemical Society. **d** Light-responsive and **e** pH-responsive dynamic plasmonic switching between a relaxed state or left-/right- handed version based on two DNA origami bundles templates. Reproduced with permission from refs. [114] and [116]. Copyright 2016 American Chemical Society and 2015 Nature Publishing Group. **f** Two and **g** three AuNRs plasmonic walking on DNA origami template. Reproduced with permission from Refs. [117] and [118]. Copyright 2017 Wiley-VCH and 2018 American Chemical Society

In this context, some pioneering work was carried out by Ding and co-workers, who prepared AuNPs arranged linearly on a rectangular DNA origami sheet with precisely controlled positions and particle spacing, and then assembled them into a 3D helical geometry with chiral plasmonic phenomenon by rational rolling of the DNA origami sheet. This study opened up the possibility of realizing programmable 3D plasmonic structures with desired optical properties [110]. Thereafter, Liedl and colleagues designed a 3D DNA origami nanostructure (a nanorod with around 100 nm in length) as nanotemplate to directly organize the helical arrangement of gold nanoparticles for the generation of chiral plasmonic nanostructures. The gold–DNA nanostructure exhibited defined circular dichroism and optical rotatory dispersion effects at visible wavelengths that originated from the collective plasmon–plasmon interactions of the nanoparticles positioned with an accuracy better than 2 nm (Fig. 10a) [111]. Moreover, Wang and co-workers constructed anisotropic gold nanorod (AuNR) helical superstructures with tailored chirality based on DNA origami nanostructure templates. The ‘X’ pattern of the DNA capturing strands was predesigned on both sides of a 2D DNA origami template, and several AuNRs were modified on the origami template by base pairing, and assembled into AuNRs helices with the origami intercalated between neighboring AuNRs (Fig. 10b) [112]. Similarly, Liu’s group presented chiral plasmonic Au NPs based on a 3D DNA origami ring template (Fig. 10c) [113]. These left-handed and right-handed helical metal–DNA nanostructures all show characteristic circular dichroism effects.

In addition to the above-mentioned static chiral plasmonic nanostructures, Liu et al. also developed dynamic chiral plasmonic nanostructures based on DNA origami templates. For example, light-responsive azobenzene-modified DNA strands (Fig. 10d) [114] and pH-responsive DNA triplexes [115] serve as construction materials to organize plasmonic nanoparticles in three dimensions while concurrently driving the metal–DNA molecules to distinct conformational states. The dynamic behavior of AuNRs towards light and H^+^ stimuli was observed by CD signal changes. Similarly, strand displacement reactions were also applied to drive the plasmonic walking of AuNRs, which were recorded as dynamic CD signal changes (Fig. 10e) [116]. Recently, Lan et al. presented a new method for the tunable self-assembly of DNA chiral supramolecular architectures by creating a versatile DNA origami adapter (Fig. 10f) [117] and a reconfigurable chiral nanoparticle helix superstructure with fully switchable chirality (Fig. 10g) [118].

## Summary and Perspectives

This review summarizes recent progress in nanomaterials synthesis based on DNA nanostructure templates. With computer-aided design, the use of DNA synthesizers, and the rapid development of DNA nanotechnology, we can construct a large variety of DNA nanomaterials. This technique provides valuable opportunities for more controllable preparation of organic and inorganic materials, polymer materials and other synthetic materials, indicating that its influence has distinctly penetrated all aspects of material science. Mimicking the central dogma, translating DNA sequence to other synthetic polymeric materials provides a valuable method of achieving sequence-controlled polymers. Based on larger DNA nanostructures, one can confine polymer synthesis to obtain more sophisticated polymer nanostructures or anisotropic polymer films, or even control the alignment of a single polymer chain to fabricate single molecular level electronic circuits. The self-assembly of inorganic nanoparticles based on DNA nanostructure templates can also be manipulated by DNA nanotechnology to achieve precise regulation at the nanoscale, including lattice regulation, chiral regulation and dynamic growth regulation. Moreover, in situ synthesis of inorganic nanomaterials inside a DNA model or outside a DNA template has also emerged as a powerful method to achieve inorganic nanostructures with intricate details.

The structural variability and the capability of precise customization make DNA nanostructures an extremely powerful tool for improving high precision and controllable preparation techniques for nanomaterials. Undoubtedly, DNA-encoded synthesis technology will also bring more innovations in the field of chemical materials preparation, especially given the advantages in high-precision control of nanomaterial assembly and highly complex nanomaterial processing. This has broad application prospects in biomedicine, fine electronics, flexible materials and other fields. Despite their extraordinary prospects, several limitations have to be overcome before the widespread use of DNA nanostructures. Firstly, the cost of DNA materials has to be further reduced, which might be possible with advances in DNA synthesis techniques or the development of synthetic biology to allow direct production of DNA nanostructures in microorganisms; secondly, the yield and robustness of these methods have to be further proved. Since most of these methods require several steps of self-assembly and reactions, reliable scale up has been shown only rarely and further optimization is required. Thirdly, DNA nanotechnology has irreplaceable advantages in achieving elaborate nanostructures from several to hundreds of nanometers, but assembly of even larger structures is relatively inefficient. Therefore, the combination of DNA-based materials synthesis together with other nanofabrication techniques, such as lithography or 3D printing, would be essential to achieve desired materials with rational designed structure from micro- to macro-level.

Overall, the development of DNA-programmed material synthesis is just beginning, and extensive efforts are still required. However, along with increasing knowledge about structure–function relationships in materials, improving the precision of nanofabrication techniques for advanced material synthesis is apparently in highly demand. Therefore, one can envision that this field will develop rapidly in the next few years. The first application breakthroughs might be in medicinal and healthcare materials, considering their favorable biocompatibility and relatively high cost.
